# A national precision cancer medicine implementation initiative for Finland

**DOI:** 10.2340/1651-226X.2024.32661

**Published:** 2024-05-23

**Authors:** Katriina J. Jalkanen, Erika Alanne, Sanna Iivanainen, Okko-Sakari Kääriäinen, Minna Tanner, Annika Auranen, Jussi Koivunen, Timo K. Nykopp, Pia Vihinen, Mika Mustonen

**Affiliations:** a Comprehensive Cancer Center, Helsinki University Hospital, Helsinki, Finland; b Turku University Hospital and FICAN West Cancer Centre, Turku, Finland; c Department of Oncology and Radiotherapy, Oulu University Hospital, Oulu, Finland; d Department of Oncology, Kuopio University Hospital, Kuopio, Finland; e TAYS Cancer Center, Tampere University Hospital and Tampere University, Tampere, Finland; f FICAN Mid, Central Finland Cancer Center, Tampere University and Tampere University Hospital, Tampere, Finland; g FICAN North, Northern Finland Cancer Center, Oulu University Hospital and University of Oulu, Oulu, Finland; h FICAN East, Eastern Finland Cancer Center, University of Eastern Finland and Kuopio University Hospital, Kuopio, Finland; i FICAN South, Southern Finland Cancer Center, Helsinki University Hospital and University of Helsinki, Helsinki, Finland

**Keywords:** FINPROVE, DRUP-Like-Trials, Precision Medicine, PRIME-ROSE, PCM4EU

To the Editor – The adaptation of nationwide genomic profiling and personalized cancer therapy in Finland has been challenging due to the lack of a uniform framework and funding. Meanwhile, increasing evidence demonstrates a clear benefit from precision medicine in cancer care [1, 2] while the need for more efficient therapies in hard-to-treat cancers is evident. Equally a challenge for these cancers remains in the lack of randomized trials.

Work to implement precision cancer medicine at a national level in Finland, began in 2021 led by Helsinki University Hospital (HUS) and FICAN South to meet three goals: (1) to implement genomic profiling and precision cancer medicine as standard of care, (2) establish equal access to molecular diagnostics and clinical trials in precision medicine, (3) to increase the number of precision cancer medicine trials and open a national DRUP (Drug Rediscovery Protocol)-like clinical trial.

A multidisciplinary **study team** including oncologists, hematologists, gynecological oncologists, pathologists, cancer researchers, molecular and clinical geneticists was formed. This nationwide working group had the common aim to enable access to personalized cancer therapy for all patients in Finland irrespective of their residence. Early discussion amongst this initiative urged the need to equally engage stakeholders for reimbursement and sustained funding. Of equal importance was to explore the possibility of a public-private partnership for drug access and possibilities for shared pay for benefit funding. Political and financial support have revealed to be the most challenging steps within this initiative as consistent public funding is still lacking. Without international coordination with other major precision cancer medicine initiatives, especially the Dutch DRUP trial, the implementation would have been impossible.

Tertiary care for cancer treatment in Finland is centralized to five university hospitals each governing a capture area of 0.7 to 1.7 M inhabitants. Each University hospital has a **regional cancer center** (FICAN South, West, East, North and Mid) that aims to promote equal access to diagnostics and treatment and, thus, the FICANs have played a major role in the precision cancer medicine initiative. During the past 3 years, we have therefore built and raised funding for an ecosystem that includes four major working groups: molecular diagnostics, data storage and secondary use, reimbursement, and finally the national DRUP like trial. National infrastructure for precision cancer diagnostics requires all five university hospitals across Finland, and these in turn facilitate the use of advanced molecular diagnostics, operate local Molecular Tumor Boards (MTBs) and participate in our national MTB. The national MTB operates digitally (Teams) on a weekly basis evaluating cases that have been pre-screened for FINPROVE by the local MTBs (Figure 1).

**Figure 1:**
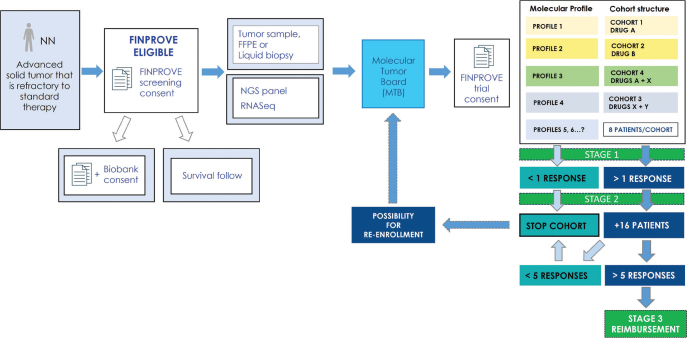
FINPROVE design and patient inclusion.

To facilitate precision cancer medicine implementation and patients’ access to targeted anti-cancer drugs, FINPROVE was launched in August 2021. FINPROVE is a national investigator-initiated interventional phase 2 trial that opened for inclusion in January of 2022 first in Helsinki University Hospital, and now the trial is open in all five University Hospitals. Smaller regional hospitals operate through their governing university hospital to include patients in FINPROVE. The trial is coordinated with and modeled on, the Dutch DRUP trial, which has been successful in the uptake of precision medicine nationwide, due to high inclusion rates resulting in significant patient benefit and exploring new national reimbursement models [3, 4]. FINPROVE is also aligned with similar trials in Denmark (ProTarget), Norway (IMPRESS) and Sweden (FOCUSE). Importantly, EU has funded two large precision cancer initiatives facilitating collaboration within countries working on these trials and precision medicine: PCM4EU and PRIME-ROSE. Both initiatives comprise of projects for research on control cohorts, use of real-world evidence, reimbursement models and health economics, ethics, legal aspects, and data governance. Partly due to this, our national initiative has gained substantial interest in patient organizations, private companies and stakeholders involved in reimbursement strategies for new therapy/drug indications. The majority of funding is covered through the University Hospitals but of equal importance have been private trusts. Additional funding comes from company contributions for drugs, and costs per-patient in FINPROVE.

The FINPROVE ecosystem is currently scaling to include regional hospitals as sites in aiming at nationwide equality for both molecular profiling and inclusion to precision medicine trials. The major challenge for larger uptake of genomic profiling is the lack of public reimbursement for advanced molecular panels and the diversity of genomic analysis used. For this reason, access to expensive panels has not been uniform and requires ongoing discussions with public stakeholders as agnostic indications and approved targeted agents have entered standard-of-care with increasing pace. Actionable targets are confirmed centrally upon trial entry. Yet for screening all University Hospitals use commercial panels (e.g. TruSight Oncology 500, Foundation One Cdx, Oncomine Comprehensive Assay Plus) while validating in-house panels that will hopefully be available for all cancer patients in Finland in the future as national guidance for screening still requires implementation.

By November 2023, the national MTB has evaluated 450 patients with molecular profiling results for inclusion in the FINPROVE trial and other ongoing studies. Of these, more than 100 (25%) patients have been offered treatment within FINPROVE which consists of cohorts defined by a specific molecular profile (eg. mutation, fusion, or amplification) and drug. The trial is a cluster of cohorts that follows a Simon two-stage model with Stage 1 consisting of a cohort of eight patients in a combined umbrella and basket design. Positive cohorts (≥5 of 24 responsive patients, Stage 2) may expand into larger cohorts (Stage 3) from which discussions on drug reimbursement can be based on. Currently reimbursement is only case-based and models for extension of indications are to be explored. When the trial started, eight drugs were offered from Roche, but continuing discussions with other companies have resulted in the inclusion of drugs from Bayer, Novartis, Eli Lilly, and Janssen, adding up to 13 drugs at the end of 2023. By the end of the second quarter in 2024, we foresee to have additional companies, and 18 drugs in the trial, improving patient inclusion and, hopefully, benefits for the patients.

Through this initiative, we now have an ecosystem that has a mutual aim to increase clinical trial access for cancer patients across Finland, harmonize molecular testing, and equalize standard-of-care. Patients with progressive cancer disease can now be referred to their governing University Hospital irrespective of where they live and have the possibility for advanced molecular cancer diagnostics [5]. Through the national MTB, an increasing number of cancer patients now have access to more treatment lines, both for standard-of-care and experimental drugs, beyond previous availability. The whole ecosystem strengthens translational research and innovation through extensive data generation, biobanking and national registries. All University Hospitals have the competence to utilize their data lakes to promote drug efficacy data, data on health economics for future technology assessments, new diagnostic methods and re-enforce international competence through AI.

Future plans for this precision cancer medicine ecosystem include expanding the drugs included in FINPROVE while also allowing drug combinations especially for hematologic malignancies. Moreover, a deeper understanding on the molecular underpinnings of response and resistance of tumors to targeted treatments is urgently needed to enhance precision oncology approaches as it is unknown why some of the patients with the same molecular alteration respond and some fail to respond to the same therapy [6].

**FINPROVE PIs** Katriina Jalkanen^1^, Erika Alanne^2^, Sanna Iivanainen^3^, Okko Kääriäinen^4^, Minna Tanner^5^.

**FINPROVE Study Group**: Katriina Jalkanen^1^, Erika Alanne^2^, Minna Tanner^5^, Okko Kääriäinen^4^, Sanna Iivanainen^3^, Annika Auranen^6^, Anniina Färkkilä^1,11^, Heini Lassus^1^, Peeter Karihtala^1^, Marjukka Pollari^5^, Sanni Tulokas^1^, Siru Mäkelä^1^, Sari Jäämaa^1^, Jarkko Ahvonen^5^, Justiina Kaukola^1^, Timo Makkonen^1^, Laura Kohtamäki^1^, Milla Katajisto^1^, Emmi Peurala^1^, Päivi Halonen^1^, Synnöve Staff^13^, Satu Karjalainen^1^, Inga Bauer^1^, Heidi Burmoi^5^, Jenna Renko^5^, Nina Halme^2^, Riina Sonkkila^2^, Marjo Väätäinen^4^, Raija Mikkonen^3^, Jessica Hannelius^5^, Kirsi Penttilä^5^, Tanja Juslin^1^.

^11^Research Program in Systems Oncology & Department of Obstetrics and Gynecology, University of Helsinki and Helsinki University Hospital; ^13^Department of Obstetrics and Gynecology, Tampere University Hospital and Faculty of Medicine and Health Technology, Tampere University, Tampere, Finland.

**FINPROVE Molecular Tumor Board:** Katriina Jalkanen^1^, Erika Alanne^2^, Minna Tanner^5^, Okko Kääriäinen^4^, Anniina Färkkilä^1^, Heini Lassus^1^, Peeter Karihtala^1^, Ari Ristimäki^14^, Annukka Pasanen^12^, Soili Kytölä^15^, Reetta Vainionpää^15^, Elina Niemelä^15^, Elina Kaikkonen^15^, Iiris Ukkola^15^, Riikka Nurminen^16^, Minna Pöyhönen^15,17^, Nelli Saijets^15,17^, Mauri Keinänen^16^, Veli Kairisto^18^, Eva-Maria Talvitie^18^, Tanja Juslin^1^, Satu Karjalainen^1^.

^14^Department of Pathology, HUSLAB, HUS Diagnostic Center, University of Helsinki and Helsinki University Hospital, Helsinki, Finland; ^15^HUSLAB, Department of Genetics, HUS Diagnostic Center, Helsinki University Hospital and University of Helsinki, 00029 Helsinki, Finland; ^16^Department of Genetics, Fimlab Laboratories, Tampere, Finland; ^17^Department of Medical and Clinical Genetics, University of Helsinki, Helsinki, Finland; ^18^Department of Genomics, Turku University Hospital, Turku, Finland.

**Translational team**: Anniina Färkkilä^1,11^, Soili Kytölä^15^, Sirpa Leppä^19^, Ari Ristimäki^14^, Antti Rannikko^20^, Anni Virtanen^14^.

^19^Applied Tumor Genomics, Research Programs Unit, Faculty of Medicine, University of Helsinki, Helsinki, Finland; Department of Oncology, Helsinki University Hospital Comprehensive Cancer Center, Helsinki, Finland; ^20^Department of Urology and Research Program in Systems Oncology, University of Helsinki, and Helsinki University Hospital, Helsinki, Finland

**Regional FICANs**: Annika Auranen^6^, Jussi Koivunen^7^, Mika Mustonen^10^, Timo Nykopp^8^, Pia Vihinen^9^

## Data Availability

The trial is on progress.
